# Change in the Ipsilateral Motor Cortex Excitability Is Independent from a Muscle Contraction Phase during Unilateral Repetitive Isometric Contractions

**DOI:** 10.1371/journal.pone.0055083

**Published:** 2013-01-31

**Authors:** Kazumasa Uehara, Takuya Morishita, Shinji Kubota, Kozo Funase

**Affiliations:** 1 Human Motor Control Laboratory, Division of Human Sciences, Graduate School of Integrated Arts and Sciences, Hiroshima University, Higashi-Hiroshima, Hiroshima, Japan; 2 Research Fellow of the Japan Society for the Promotion of Science, Tokyo, Japan; University of Toronto, Canada

## Abstract

The aim of this study was to investigate the difference in a muscle contraction phase dependence between ipsilateral (ipsi)- and contralateral (contra)-primary motor cortex (M1) excitability during repetitive isometric contractions of unilateral index finger abduction using a transcranial magnetic stimulation (TMS) technique. Ten healthy right-handed subjects participated in this study. We instructed them to perform repetitive isometric contractions of the left index finger abduction following auditory cues at 1 Hz. The force outputs were set at 10, 30, and 50% of maximal voluntary contraction (MVC). Motor evoked potentials (MEP) were obtained from the right and left first dorsal interosseous muscles (FDI). To examine the muscle contraction phase dependence, TMS of ipsi-M1 or contra-M1 was triggered at eight different intervals (0, 20, 40, 60, 80, 100, 300, or 500 ms) after electromyogram (EMG) onset when each interval had reached the setup triggering level. Furthermore, to demonstrate the relationships between the integrated EMG (iEMG) in the active left FDI and the ipsi-M1 excitability, we assessed the correlation between the iEMG in the left FDI for the 100 ms preceding TMS onset and the MEP amplitude in the resting/active FDI for each force output condition. Although contra-M1 excitability was significantly changed after the EMG onset that depends on the muscle contraction phase, the modulation of ipsi-M1 excitability did not differ in response to any muscle contraction phase at the 10% of MVC condition. Also, we found that contra-M1 excitability was significantly correlated with iEMG in all force output conditions, but ipsi-M1 excitability was not at force output levels of below 30% of MVC. Consequently, the modulation of ipsi-M1 excitability was independent from the contraction phase of unilateral repetitive isometric contractions at least low force output.

## Introduction

Recent transcranial magnetic stimulation (TMS) studies in human subjects, we found an increase in motor evoked potential (MEP) induced in the resting hand muscle contralateral to movement side, which reflects ipsilateral primary motor cortex excitability (ipsi-M1), while performing a unilateral rhythmic voluntary abduction of the index finger [1] or a fine-motor manipulation task [2], [3] with weak muscle contractions (around 10 to 15% of maximal voluntary contraction, MVC). Among them, we observed that MEP induced in the contralateral resting hand did not depend on the unilateral muscle contraction phase, as assessed by using analysis of correlation between the MEP induced in the contralateral resting hand and the electromyogram (EMG) activity in the active muscles for the 100 ms preceding TMS onset [1]–[3]. However, further direct evidence on the effect of muscle contraction phase dependent on the modulation of ipsi-M1 excitability during a performance of unilateral movement should be investigated to establish the systematic method for evaluation of the ipsi-M1 excitability using the TMS paradigm.

The background of the modulations of ipsi-M1 excitability induced during unilateral movement shows that M1 in the both hemispheres are activated and interfere with each other. This interference is considered to be mediated by the transcallosal pathway [4], [5]. Indeed, it has been reported that ipsi-M1 excitability is altered by task-related modulation during unilateral upper limb movement at relative weak muscle activation in monkeys [6]–[8]. Likewise, a human TMS study performed during various hand motor tasks found not only changes in contralateral M1 (contra-M1) excitability, but also changes in ipsi-M1 excitability, confirming the complex mechanism involved in their activation. In particular, motor tasks requiring a high degree of dexterity, rhythmic repetitive hand movements or sustained isometric contraction at relative weak muscle activity have been demonstrated to markedly enhance ipsi-M1 excitability [1]–[3], [9]–[12]. These studies suggest that the effect of performing a unilateral hand motor task on change in the ipsi-M1 excitability can be attributed to the transcallosal pathway. In addition, functional magnetic resonance imaging (fMRI) studies have also reported the deactivation of the ipsi-M1 during the performance of a unilateral finger motor task [13], [14].

The relationships between the performance of a phasic unilateral hand motor task and changes in ipsi-M1 excitability have been investigated in several studies [15]–[18]. In particular, Carson [17] investigated the movement phase dependence of ipsi-M1 excitability during the performance of a unilateral wrist movement referring to a phase angle within a sinusoidal cyclic pattern with dynamic joint movements. We predict that when subjects perform such movements, the muscle contraction levels of the agonist and antagonist muscles vary erratically. Indeed, Carson’s study indicated that the root mean square EMG levels of the wrist muscles during execution of phasic wrist flexion and extension movement ranged from 1.3% to 33.5% of MVC, and it seems likely that this study mainly focus on the relationship between the phase-dependent modulation of ipsi-M1 excitability and such dynamic joint movement phase rather than a muscle contraction. Therefore, we consider that the change in ipsi-M1 excitability induced during the unilateral phasic wrist muscles contraction might be affected by the unsteady muscle force output change in the agonist and antagonist muscles. Hence the previous study as described above might be not able to completely establish the effect of muscle contraction phase-dependent on the change in ipsi-M1 excitability. Howatson et al. [19] investigated the effects of different types of muscle contraction on the change in ipsi-M1 excitability. As a result, they demonstrated that ipsi-M1 excitability was significantly greater during lengthening contractions than shortening contractions. Another study suggested that despite the same level of background EMG activity being recorded during shortening and lengthening contractions, size of the MEP induced in response to TMS was significantly greater during shortening contractions than lengthening contractions [20]. Thus, differences in the type of muscle contraction affect the changes in ipsi-M1 excitability. Hence, it is important to investigate the effect of the phase dependence based on the muscle contraction property on change in the ipsi-M1 excitability. To achieve our purpose, we employed a simple repetitive isometric contraction without any dynamic joint movements. Thus, the aim of this study was to further investigate whether ipsi-M1 excitability depends on the muscle contraction phase of repetitive isometric contractions with unilateral index finger abduction and whether this phenomenon is affected by the force output level of repetitive isometric muscle contractions. We conducted the following series of experiments. In experiment 1, we examined whether the changes in MEP amplitude in the active or resting first dorsal interosseous (FDI) are dependent on the timing of the TMS trigger to the contra (active)- and ipsi (resting)-M1 during unilateral repetitive isometric contractions of the FDI. In experiment 2, we examined the relationship between the changes in MEP amplitude evoked in the active or resting FDI by randomly triggered TMS and the integrated EMG (iEMG) in the active FDI for the 100 ms preceding TMS onset in the same way as in experiment 1. We hypothesized that the changes in MEP amplitude in the active FDI, which represent contra-M1 excitability, would be affected by the muscle contraction phase during repetitive isometric contractions, but that those in the resting FDI, which represent ipsi-M1 excitability, would not be affected by any muscle contraction phases.

## Materials and Methods

### Subjects

Ten healthy volunteers (five females, age range: 20–24 years) participated in this study, all of whom were right handed, as assessed using the Edinburgh Handedness Inventory [21], and gave their written informed consent. All experimental procedures were carried out in accordance with the Declaration of Helsinki and approved by the local ethics committee at Hiroshima University.

### EMG and Force Output Recordings

The subjects were comfortably seated on a reclining chair and instructed to put both hands on a horizontal plate attached to the chair’s armrests. Surface EMG activity was recorded from the right and left FDI using 9 mm diameter Ag-AgCl surface cup electrodes. The EMG activity was filtered at a bandwidth of 5 Hz to 3 kHz, and all amplification procedures were controlled using a signal processer (model 7S12, NEC San-ei Co. Ltd., Japan). The analog outputs from the signal processor were digitized at a sampling rate of 10 kHz and saved on a computer for off-line analysis (PowerLab system, AD Instruments Pty., Ltd., Australia). Prior to the beginning of the experiment, we measured the force output of left index-finger abduction at MVC against an immobile bar with a force sensor to provide a reference value for each individual, and then we calculated the 10, 30, and 50% of MVC values for each individual. We loosely fixed each subject’s left index finger to the immobile bar using a rubber band to maintain contact between the index finger and the force sensor. The force signal was amplified by a strain amplifier (model 6M82, NEC San-ei Co. Ltd., Japan), which was connected to the force sensor. Each subject's force output was displayed on an oscilloscope monitor, which was placed approximately 1 m in front of the subject. Two lines were shown on the monitor, one representing the force output being generated by the individual being tested and the other representing the target force output (10, 30, 50% of MVC) for each individual.

### Experimental Design

In experiment 1, we instructed the subjects to perform repetitive isometric contractions of left index finger abduction at 10% of MVC, as precisely as possible upon hearing an isochronous auditory cue, which consisted of duration of 100 ms and monotone burst, delivered at 1 Hz (one beat per muscle contraction and relaxation) and to relax their right finger and forearm throughout all of the experiments. TMS was automatically delivered to the M1 at one of eight intervals after EMG onset (see “TMS” and “TMS trigger”). In experiment 1, the force output was only set to 10% of MVC because we preliminarily confirmed that performing repetitive isometric contractions at 30 or 50% of MVC conditions throughout sessions for experiment 1 provoked heavy muscle fatigue due to keeping of a large number of muscle contractions during each session. Hence, we excluded 30 and 50% of MVC from experiment 1. On the other hand, the experiment 2 was not observed any muscle fatigues provoked by high force output conditions (i.e., 30% and 50% MVC) due to small number of muscle contractions during each session than the experiment 1.

**Figure 1:**
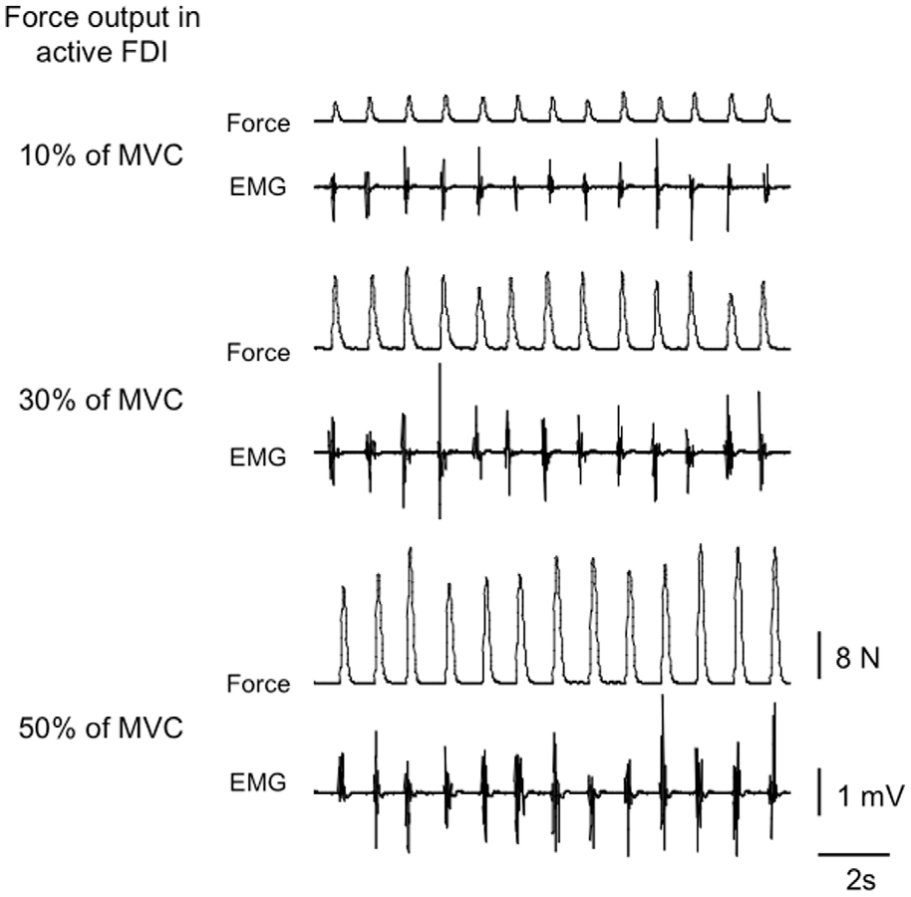
Typical EMG and force output traces. Each trace shows examples of force and EMG in the left FDI and the level of each force in a typical subject who performed repetitive contractions of their unilateral index finger at different force output levels upon hearing an auditory cue delivered at 1 Hz.

In experiment 2, we instructed the subjects to perform repetitive isometric contractions of left index finger abduction at three force output levels (10, 30, 50% of MVC) as precisely as possible upon hearing an auditory cue delivered at 1 Hz in the same way as mentioned above. TMS delivered to each M1 were randomly triggered regardless of the contraction phase in the left FDI (see “TMS” and “TMS trigger”). Figure 1 shows the force output and EMG activity in each condition. The subjects were sufficiently given a rest break between each trial to avoid a finger muscle fatigue. In addition, in order to record MEP in each FDI muscle at the control condition (i.e., both muscles resting condition), we asked the subjects to completely relax their both finger and forearm muscles then TMS were delivered to each M1 at 7–10 sec intervals. Twelve MEPs were recorded from each FDI muscle. The order of the force output and resting conditions was randomized for each subject to avoid order effects.

### TMS

Magnetic stimulation was delivered over the M1 in the hemisphere using a Magstim 200 stimulator (Magstim Co. Ltd., UK) with a figure-of-eight shaped coil with an external diameter of 90 mm, which was placed over the M1 tangential to the scalp with the handle of the coil pointing backwards and rotated approximately 45° away from the midsagittal line. We found the optimal coil position for evoking MEP in each FDI by moving the coil in 1 cm steps around the presumed hand motor area within the M1. The site at which stimulation with a slightly suprathreshold TMS intensity consistently evoked the largest MEP in each FDI was regarded as the motor hotspot and was marked with a pen on a swimming cap covering the subject’s scalp. The resting motor threshold (rMT) was defined as the lowest stimulus intensity that evoked MEP in each FDI at an amplitude of at least 50 µV in five out of ten trials. The test stimulus intensity was carefully adjusted to elicit an MEP peak-to-peak amplitude of around 1 mV in each FDI at the control condition (i.e., both muscles resting condition). The rMT (mean ± standard deviation) for left and right M1 for all of subjects were 48.7±5.0 and 49.9±6.9% of maximal stimulator output, respectively.

### TMS Trigger

We deliberately changed the TMS trigger method between experiments 1 and 2. In the experiment 1, TMS were automatically delivered to the left and right M1 in alternating blocks at eight intervals (0, 20, 40, 60, 80, 100, 300, or 500 ms) after the EMG onset when each interval had reached the triggering slice level, which was set at an EMG activity of 500 µV in the active left FDI. In the preliminary experiment, we confirmed that this slice level generates a stable triggering for TMS at ascending phase of ballistic EMG activity during isometric muscle contractions of left FDI. We recorded 7–10 MEP that was delivered at intervals of 7–10 sec according to the setup TMS trigger level in each of the eight intervals. During the each interval, the subjects continued performing the repetitive isometric contraction according to auditory cue at 1 Hz. In the experiment 2, the TMS delivered to the M1 at intervals of 7–10 sec was randomly triggered during all phases of the repetitive index finger abduction of each FDI; i.e., we purposefully ignored whether the TMS was delivered during the ascending or descending phase of a raw EMG burst in order to record MEP and iEMG in a random phase. Stimulus to right and left M1 were randomly intermixed and we recorded 30 MEP at each condition in the both M1, respectively. During the each trial, the subjects continued performing the repetitive isometric contraction according to auditory cue at 1 Hz with taking an enough rest.

### Data and Statistical Analyses

MEP amplitude was analyzed using peak-to-peak values, which are expressed as a percentage of the mean MEP amplitude in the control (resting) conditions. In experiment 2, in order to analyze the correlation between MEP and iEMG, we calculated iEMG values from a rectified EMG in the active (i.e., right) and resting (i.e., left) FDI muscles during the 100 ms window preceding the TMS trigger, using “Integral Abs, Scope version 3.7.6., Power Lab system”. iEMG in the active FDI values are expressed as a percentage of the mean iEMG of MVC for the 100 ms window preceding the TMS trigger. The mean iEMG activities in active FDI at each force output condition were analyzed by two-way repeated measures ANOVA (force output conditions × stimulation side). On the other hand, the mean iEMG in the resting FDI values expressed in unit of “mV.ms” were analyzed by one-way repeated measures ANOVA. In experiment 1, the mean MEP amplitudes of each side (resting or active) were analyzed by one-way repeated measures ANOVA. If a significant effect was detected from two-way or one-way repeated measures ANOVAs, Bonferroni’s post-hoc test was used for detailed analysis. In experiment 2, we examined the correlation between the MEP amplitude for each FDI and the iEMG in the left FDI at each force output in individual subjects using Pearson’s correlation coefficient. If EMG activity in the resting FDI (i.e., right) was detected at preceding the TMS trigger, this trial was excluded from the analysis. In all of analyses, the level of statistical significance was set at p<0.05. All values are presented as the mean ± standard error (SE).

## Results

### iEMG Activity in Resting FDI

The mean iEMG values (±SE) in resting FDI for all subjects preceding the TMS trigger at rest, 10, 30 and 50% of MVC conditions were 0.70±0.05, 0.69±0.03, 0.71±0.04 and 0.71±0.03 mV.ms, respectively. No significant difference in iEMG values among conditions were detected by one-way repeated measures ANOVA (F_3, 27_ = 0.25, p = 0.85). These iEMG values were comparable to the white noise level.

### iEMG Activity in Active FDI

**Figure 2:**
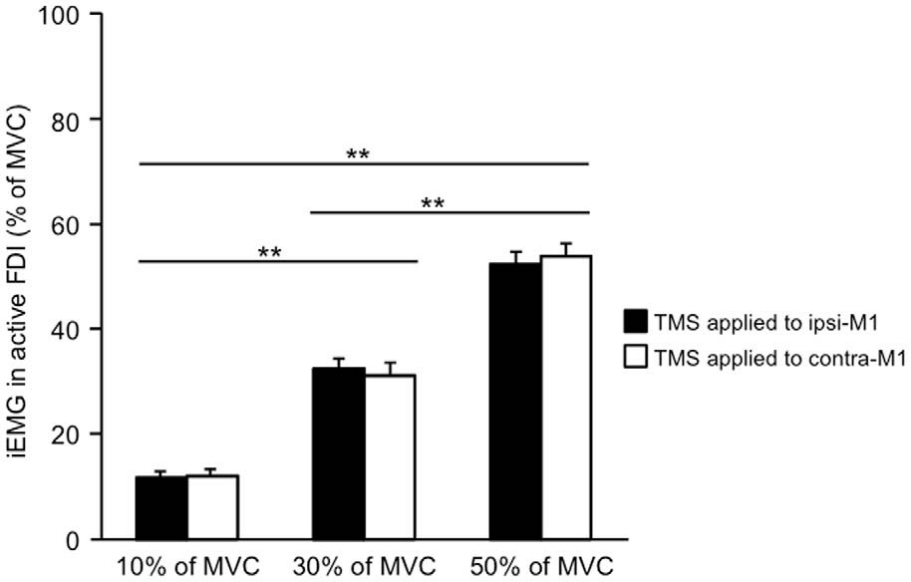
Mean iEMG activity in active FDI. Mean iEMG activity in active FDI (n = 10, ±SE) for all subjects at each condition. Each iEMG activity was obtained from the EMG of 100 ms window in active FDI prior to TMS trigger. The asterisks indicate a significant difference: ** p<0.01, * p<0.05.

Figure 2 shows the mean iEMG values in active FDI for all subjects at each condition. The two way repeated measures ANOVA detected a significant main effect of “force output conditions“ (F_2,57_ = 197.7, p<0.01). And, no significant main effect of the “stimulation side“ (F_1,57_ = 0.002, p>0.05) and no significant interaction of both factors (F_2,57_ = 0.196, p>0.05) were detected, respectively. According to further analyses using Bonferroni’s post-hoc test, the iEMG values in the left FDI were significant difference between each force output condition (p<0.01). The iEMG values in active FDI were robustly coincident with individual target force levels, suggesting that the subjects accurately performed them according to each target force throughout the experiments.

### Relationship between Changes in MEP Amplitude in the Active or Resting FDI and the EMG Onset-TMS Interval

**Figure 3:**
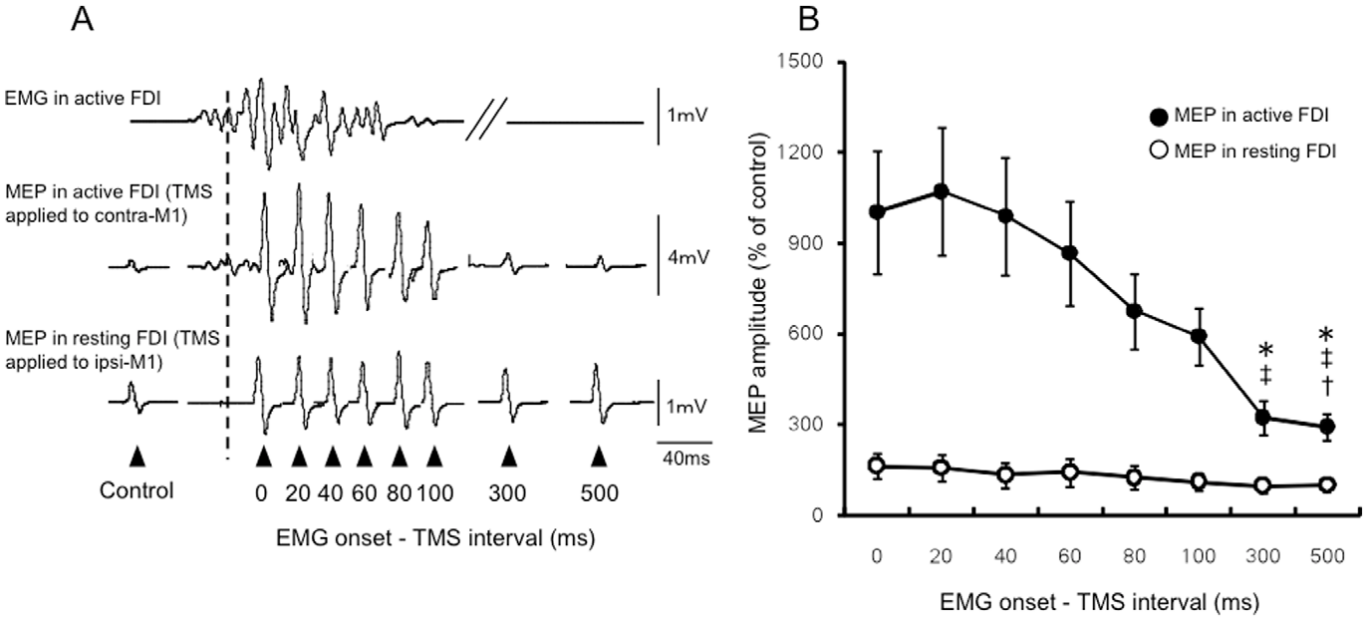
Time course of the changes in MEP amplitude (Experiment 1). (A) The traces show the typical MEP amplitudes for each FDI and EMG activity in the left FDI from one representative subject. (B) The overall mean time courses of the changes in MEP amplitude (n = 10, ±SE) evoked in the ipsi- (open circles) and contra-FDI (closed circles) muscles by a single TMS as a percentage of the control values. The asterisks indicate a significant difference from EMG onset (*p<0.05). The daggers indicate a significant difference from the 20 ms EMG onset-TMS interval (‡p<0.05) or 40 ms EMG onset-TMS interval (†p<0.05).

Figure 3A shows the EMG activity and the changes in MEP amplitude for a representative subject. The MEP amplitudes in the active FDI increased markedly and were subsequently attenuated as the EMG onset-TMS interval was prolonged. On the other hand, the MEP amplitudes in the resting FDI were enhanced as compared to MEP amplitude of the control condition, and these enhanced MEP amplitudes remained unchanged among all of EMG onset-TMS intervals. Figure 3B shows the mean changes in MEP amplitude in the active and the resting FDI for all subjects. A significant difference in the change in the MEP amplitude of the active FDI (closed circles) was detected among the “EMG onset-TMS intervals” by one-way ANOVA (F_7,72_ = 4.07, p<0.01). According to further analyses using Bonferroni’s post-hoc test, the MEP amplitudes evoked in the active FDI at 300 and 500 ms after EMG onset were significantly decreased compared with those observed at EMG onset and 20 ms after EMG onset (p<0.05). In addition, the EMG activity observed at 500 ms after EMG onset was significantly decreased compared with that observed at 40 ms after EMG onset (p<0.05). In contrast, no significant difference in the MEP amplitude of the resting FDI was detected among any of the EMG onset-TMS intervals (F_7,72_ = 0.46, p = 0.85).

### Relationship between the Change in MEP Amplitude in the Active or Resting FDI and the iEMG in the Active FDI

Table 1 shows the correlation coefficients for each subject, which were obtained from correlation analyses using Pearson’s correlation coefficient. No significant correlations between the MEP amplitude in the resting FDI and the iEMG in the active FDI were found in any subject at 10 and 30% of MVC, but the significant correlations were found in four out of ten subjects at 50% of MVC. On the other hand, significant correlations between the MEP amplitude in the active FDI and the iEMG in the active FDI were found in seven out of ten subjects at 10 and 30% of MVC. At 50% of MVC, such significant correlations were found in eight out of ten subjects. These results indicate that the MEP amplitude in the resting FDI is not affected by the iEMG in the active FDI at low force outputs. In contrast, the MEP amplitude in the active FDI is consistently dependent on the iEMG in the active FDI, regardless of the force output.

**Table 1:** Results of correlation analysis (Experiment 2).

Force output in active FDI	10% of MVC		30% of MVC		50% of MVC	
	Correlation coefficient between MEP and iEMG in the active FDI					
Subjects	MEP in resting FDI	MEP in active FDI	MEP in resting FDI	MEP in active FDI	MEP in resting FDI	MEP in active FDI
A	0.21	0.16	−0.04	−0.36*	0.56**	0.67**
B	−0.08	0.78**	0.29	0.77**	−0.23	0.71**
C	−0.30	0.02	−0.35	−0.20	−0.83	0.33
D	−0.27	0.37**	0.24	0.40*	−0.07	0.71**
E	−0.05	0.19	−0.05	0.09	−0.48	0.94
F	0.20	0.40**	0.20	0.37	−0.14	0.58**
G	0.85	0.64**	0.56	0.50**	0.52**	−0.36**
H	0.22	0.35*	0.13	0.41*	0.43**	0.55**
I	0.20	0.54**	0.26	0.75**	0.38*	0.84**
J	−0.32	0.70**	0.78	0.58**	−0.65	0.58**
Number of significant correlation coefficient	0/10	7/10	0/10	7/10	4/10	8/10

Correlation coefficients for each subject (subject A–J) in each condition (n = 10). The asterisks indicate a significant difference: **p<0.01, *p<0.05. The bottom row shows the number of significant correlation coefficients.

## Discussion

The present study was to investigate the muscle contraction phase dependence of ipsi-M1 excitability during performance of the unilateral rhythmic contractions of index finger abduction from two different perspectives. We investigated the time course of the changes in MEP amplitude in the resting FDI (i.e., ipsi-M1 excitability) during repetitive isometric contractions of index finger abduction in the experiment 1. In addition, we analyzed the correlation between the MEP amplitude in the resting/active FDI and the iEMG in the active FDI during the same task in experiment 2. We found that although the changes in MEP amplitude in the active FDI were markedly affected by the muscle contraction phase, the changes in the MEP amplitude in the resting FDI were unaffected by that of a unilateral repetitive isometric contractions of index finger abduction at low force output levels (less than 30% of MVC). Therefore, our findings suggest that the enhanced ipsi-M1 excitability induced by unilateral repetitive isometric contractions of index finger abduction was hardly affected by the muscle contraction phases at low force output levels, as compared to the much enhanced contra-M1 excitability which was evidently dependent on the muscle contraction phase.

There are two possible mechanisms that could explain this phenomenon: spinal level interaction or interaction mediating via a transcallosal pathway from the active M1 to the resting M1, because it is thought that change in MEP size depends on both the M1 and spinal motoneuron activity. Funase and Miles [22] reported that MEP amplitude showing the contra-M1 excitability was increased with increasing EMG activity during unilateral wrist movement, and it was also affected by the spinal motoneuron activity. Indeed, some reports have shown that ipsi-M1 excitability was increased at high force output levels (over 50% of MVC), which could be attributed to interactions at the spinal level [23]–[25]. Therefore, during unilateral repetitive isometric contractions in high force output conditions, spinal motoneuron activity might influence the change in MEP amplitude in the resting finger muscle. Hence, there is a possibility that the muscle contraction phase dependence of changes in ipsi-M1 excitability is increased in high force output conditions, which might involve changes in spinal motoneuron activity. In an fMRI study combined with EMG recording, a unilateral hand grip force task demonstrated that EMG activity in the hand muscles was strongly associated with activity in not only contra- but also ipsi-M1 with increasing muscles contraction level [26]. Another fMRI study demonstrated that a unilateral high force output task increased blood-oxygen-level-dependent (BOLD) signals, which reflect synaptic activity or action potentials in the brain, in not only contra- but also ipsi-M1. In contrast, during a low force output task the level of BOLD signals in the ipsi-M1 remained unchanged compared to that in the contra-M1 [27]. Given the results of these fMRI studies, higher force output levels result in the firing of many pyramidal tract neurons and interneurons within both M1 to generate descending commands to the performing muscles. Regarding the effect of the force output level on the transcallosal pathway, increasing the functional demands of a unilateral motor task, such as employing a high force output or dexterous task, modulates the propagation of the transcallosal pathway from the active to resting M1, which results in a modulation of ipsi-M1 excitability, because interhemispheric inhibition (IHI) from the active to resting M1 is decreased by unilateral high force output tasks as compared to that observed during low force output tasks [10], [28]. Another study involving the simultaneous use of an electroencephalogram and TMS has reported the phase dependence of ipsi-M1 excitability during a forceful unilateral finger movement (i.e., high force output). There is a good evidence to suggest that ipsi-M1 excitability and ipsi-event-related desynchronization were dependent on the time course of the forceful unilateral finger movement [29].

We detected a few negative correlations between MEP in resting FDI (i.e., ipsi-M1 excitability) and iEMG at 50% of MVC condition (see Table 1). One of the interpretations of this phenomenon is that change in sensitivity of the ipsi-M1 excitability to force output level might be involved. Indeed, despite the performing unilateral muscle contraction accompanying with an increase in force output, change in the ipsi-M1 excitability is not always linear modulation [23], [30]. These studies have also reported that change in the ipsi-M1 excitability at around 50% of MVC has poor sensitivity to force output compared to low force output levels. In addition, previous reports demonstrated that MEP amplitude in the hand muscle induced during the low force output is larger than those induced during high force output in human subjects [12], [31], [32]. Thus, there is a possibility that high force output condition led to the diminished sensitivity for changing in the ipsi-M1 excitability. However, it should be noted that these findings were detected from few subjects and were not main focus in the present study. Further systematic experiment will be required to reveal the relationship between sensitivity of ipsi-M1 excitability and force output level produced by the unilateral finger muscle contraction.

In early TMS studies, the discharge of single motor units following the application of TMS to the M1 was examined using the post-stimulus time histogram technique [33], [34]. These studies have demonstrated that the discharge of single motor units after TMS was increased when the subject maintained tonic contraction of their finger muscle. This was due to the cycle of membrane potential changes produced in response to the voluntary drive [33], [34]. In an animal study, it was reported that the Ia afferent inputs from the musculotendinous receptors of the hand muscles facilitate spinal motoneuron excitability just after the onset of muscle contraction [35]. Moreover, these afferent inputs are in direct contact with the sensory area and the M1 contralateral to afferent inputs side [36]. Thus, the dependence of contra-M1 excitability on the time course after unilateral muscle contraction can be attributed to not only motor commands, but also their peripheral afferent inputs. In contrast to the case for contra-M1 excitability, it is able to be suggested that changes in ipsi-M1 excitability are hardly affected by afferent inputs arising from the opposing body side. As a reason for this phenomenon, when we record the ipsi-M1 (i.e., resting M1) excitability during a performance of unilateral hand task using TMS, state of opposing homonymous hand muscle innervated by the resting M1,which is employed to record MEPs, remains in the complete resting. Consequently, changes in the ipsi-M1 excitability are not dependent on the movement phase in the performing side during unilateral finger movement at low force outputs.

In the present study, enhancement of ipsi-M1 excitability induced during the unilateral repetitive muscle contractions was observed as compared with that seen at resting condition, which was consistent with previous studies [1]–[3], [10]–[12]. However, Liepert et al. [24], who reported that a unilateral force generation task using pinch grip at exceedingly-low force output (1–2% of MVC) can provoke an inhibitory effect of ipsi-M1. As just described, changes in the ipsi-M1 excitability induced by unilateral tasks are split into inhibitory or facilitatory effect. One of the reasons for this discrepancy is that changes in the ipsi-M1 excitability induced by unilateral tasks were complex and varied between muscles, task difficulties, and all that. In addition, accustomed (e.g., pinch grip) or less-accustomed task (e.g., repetitive isolated movement of index finger abductions) with use of unilateral finger might modulate the inhibitory/facilitatory balance within the ipsi-M1. Rau and colleagues [29] made a point that relative less-accustomed tasks appear to elicit more facilitation than accustomed or very well practiced tasks.

### Conclusions

This study examined whether the change in ipsi-M1 excitability depends on the muscle contraction phase in the active FDI during unilateral repetitive isometric contraction of index finger abduction. Despite the remarkable changes in contra-M1 excitability observed at different EMG onset-TMS intervals during the finger movement task at 10% of MVC, ipsi-M1 excitability was not affected by the EMG onset-TMS interval. In addition, no correlation between the MEP amplitude in the resting FDI and EMG in the active FDI was found in the 10 or 30% of MVC conditions, but a correlation between these variables was found at 50% of MVC. In conclusion, the change in ipsi-M1 excitability is not dependent on the muscle contraction phase at low force outputs (below 30% of MVC) during repetitive isometric contractions of a unilateral finger muscle.
